# The Lateral Para-Patellar Approach for Intramedullary Tibia Nailing in Distal Tibia Extra-articular Fractures: A Prospective Cohort Study

**DOI:** 10.7759/cureus.62940

**Published:** 2024-06-23

**Authors:** Jitendra Mishra, Sunit Pani, Tapan Das, Chaitanya Khandelwal, Sourav Mishra

**Affiliations:** 1 Department of Orthopaedics, Institute of Medical Sciences and SUM Hospital, Bhubaneswar, IND

**Keywords:** extra-articular distal tibia fractures, intramedullary tibial nailing, extraarticular approach, semiextended nailing, lateral parapatellar approach

## Abstract

Background and objectives: The treatment of extra-articular distal tibia fractures is still a subject of debate and frequently necessitates surgical treatment, and intramedullary nailing (IMN) offers a minimally invasive approach with excellent results. Important factors in these procedures are positioning, operative duration, and radiation exposure. This study details the semi-extended lateral para-patellar approach for IMN of distal tibia extra-articular fractures and documents our findings regarding operative time, intra-operative radiation exposure, residual anterior knee pain, knee functional and radiological outcomes at six months follow-up.

Methods: We reviewed the cases of 60 patients who underwent IMN for distal tibia extra-articular fractures from May 2022 to March 2024, employing an extra-articular lateral para-patellar approach in the semi-extended position. Patients were evaluated clinically and radio-graphically for a minimum follow-up period of six months. Data collected included duration of surgery, intraoperative radiation exposure, and knee functional score for all patients. Assessment of fracture healing, residual deformities, residual anterior knee pain, and range of motion of the treated knee compared to the contralateral knee was done at a six-month follow-up.

Results: The average surgery duration was 54 ± 5 minutes, with intraoperative imaging averaging 48 exposures. The average time to union was 16 ± 3 weeks. Six months post-surgery, the mean Knee Society Score was 86.4 ± 3.5 (out of 100). At the six months follow-up, all patients exhibited clinical and radiographic healing, with only two cases showing mal-alignment (angular deformity <10 degrees). All patients regained a comparable range of motion in their knees.

Conclusions: The semi-extended lateral para-patellar approach for nailing of distal tibia extra-articular fractures enhances reduction, simplifies nail insertion, reduces both fluoroscopy and operative time, minimizes anterior knee pain and improves knee functional outcomes at six months follow-up.

## Introduction

Distal tibia fractures account for 7%-10% of lower extremity fractures. The tibia, being the most commonly fractured long bone, is particularly vulnerable due to its subcutaneous position and limited soft tissue coverage, especially in the distal third [1-4]. About 85% of lower third tibia fractures are accompanied by a fibular fracture [3,4], making these fractures inherently unstable and prone to complications such as delayed union, mal-union, soft tissue issues, and infections [5-8].

The best approach for managing extra-articular distal tibia fractures remains uncertain, with no definitive treatment guidelines. Traditionally, closed lower tibia fractures have been treated with closed reduction and casting, while other methods include plating (both open and minimally invasive), interlocking nailing, and external fixation devices like the Ilizarov circular ring fixator. Each treatment modality has various technique variations.

Recently, there has been a shift towards plating both extra-articular distal tibia fractures and the associated fibular fractures. However, due to the anatomical characteristics of the distal tibia and the presence of swelling and blistering in acute trauma cases, plating often leads to soft tissue complications, including superficial infections, wound dehiscence, delayed healing, and exposed implants that may require flap coverage [9].

Intramedullary nailing (IMN) is the preferred treatment for tibial shaft fractures in adults, offering excellent functional outcomes and high union rates. However, angular mal-alignment in the frontal plane is a frequently reported complication with IMN in distal tibia fractures due to wide medullary cavity besides delayed union, mal-union, and implant failure [10]. Over the years, various surgical approaches for tibial IMN have been developed. The infra-patellar approach, first described by Küntscher in the 1940s, remains widely used. This technique involves inserting the nail into the proximal tibia through or around the patellar tendon with the knee flexed at 90 degrees [11].

Since then, other techniques have been introduced for nailing in an extended or semi-extended position. These include the para-patellar approach, which allows for extra-articular nail insertion laterally or medially to the patellar tendon [12,13], and the supra-patellar approach, which involves inserting the nail through the quadriceps tendon [14]. These semi-extended techniques, which avoid knee flexion, help neutralize displacing forces in the proximal tibia and provide better limb exposure, thereby enhancing fracture reduction options.

Semi-extended nailing was originally proposed to optimize patient positioning, fracture reduction, fluoroscopic assessment, and implant insertion. Additionally, a semi-extended approach may reduce the incidence of anterior knee pain, one of the most common complications of tibial nailing.

With the intent of avoiding intra-articular injury to the knee, Kubiak et al. described a novel technique in 2010 using the semi-extended position while performing an extra-articular lateral para-patellar approach during tibial nail placement [15].

The aim of this study was to critically analyse our experience using the lateral para-patellar approach in a semi-extended position for distal tibia extra-articular fractures by designing a prospective cohort study to document our findings regarding operative time, intra-operative radiation exposure, residual anterior knee pain, knee functional, and radiological outcomes at six months follow-up.

## Materials and methods

Study design

A single-centre prospective cohort study was conducted from May 2022 to March 2024 at the Department of Orthopedics at the Institute of Medical Sciences and Sum Hospital, Bhubaneswar, India, focusing on adults with distal tibia extra-articular fractures.

Ethical considerations

Ethical approval was obtained from the Institutional Ethics Committee on 24/04/2022 (IEC/IMS.SH/SOA/2022/695A). Additional written informed consent was obtained from all study participants for the purpose of this study, and clinical outcome data were prospectively collected on all patients.

Study criteria

Inclusion criteria comprised adults aged 18 and above with closed distal tibia extra-articular fractures (Arbeitsgemeinschaft für Osteosynthesefragen/Orthopedic Trauma Association (AO/OTA) types 43-A1, 43-A2, 43-A3) with fracture line being within 5 cm from the ankle joint, while exclusion criteria encompassed patients under 18, those with open fractures, pathological fractures, poly-trauma cases, bilateral injuries, prior knee surgery or injury, patellar instability, and those lost to follow-up.

Tools and techniques

Sixty eligible patients underwent treatment with semi-extended lateral para-patellar IMN during the study period. Data collection included patient demographics, comorbidities, injury mechanism, surgery duration, and intraoperative fluoroscopic images. Fluoroscopic radiation exposure was quantified by the number of intraoperative X-rays.

Assessments

Follow-up assessments at six weeks, three months, and six months included leg radiographs to monitor healing, alignment, and evaluations of knee range of motion (ROM) using a goniometer and comparison with contralateral knee. Clinical evaluation at follow-up included superficial or deep infection, anterior knee pain using the visual analogue scale (VAS) scoring system, objective knee function with the Knee Society Scoring system, knee stability, and quadriceps strength according to the Oxford scale.

Statistical analysis

The mean and standard deviation were used to describe the continuous variables and the frequencies and percentages for the categorical variables.

Surgical technique

The technique we used has been developed by Kubiak et al. [15] based on that originally described by Tornetta and Colins [12], and demonstrated in the surgical video technique recently presented by McAndrew et al. [16].

Although described as either medial or lateral, to standardize the procedure as much as possible, we routinely use an extra-articular semi-extended with a lateral para-patellar approach. We find it fairly easy to subluxate the patella over the medial femoral condyle in most patients and find it to be more comfortable for the surgeon to work on the lateral side of the injured limb.

A preoperative assessment for patellar instability was done, which showed stable patella in all patients. Surgeries were performed under general or spinal anaesthesia.

**Figure 1:**
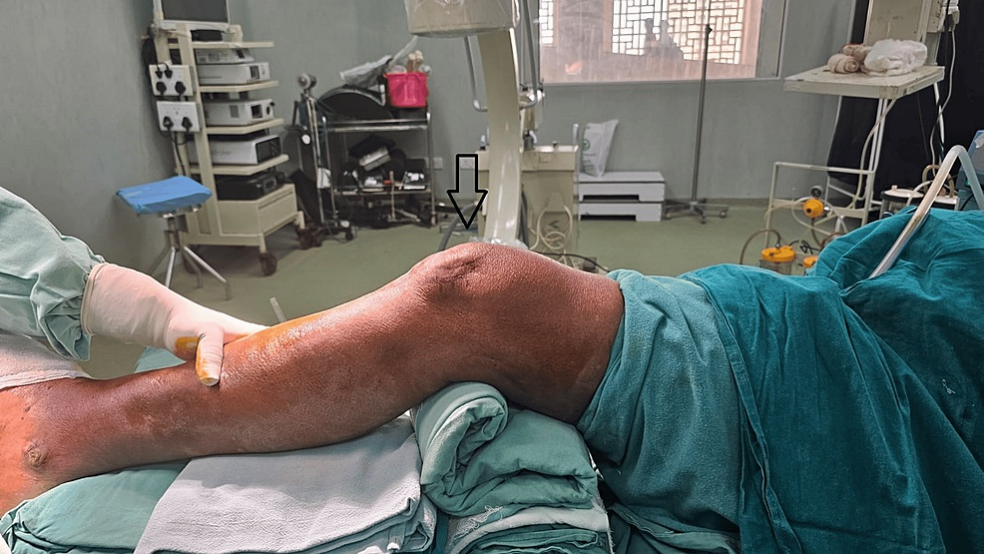
The patient is positioned supine with the leg semi-extended and the knee flexed at 20-30 degrees.

The patients were supine, and the injured leg was elevated using support underneath with 20-30 degrees of knee flexion to help take lateral X-rays and make the entry point more accessible. Anatomical landmarks were placed, and a skin incision was marked lateral to the lateral border of the patella in a curve shape inferiorly downward and lateral to the patellar tendon (Figure 1).

**Figure 2:**
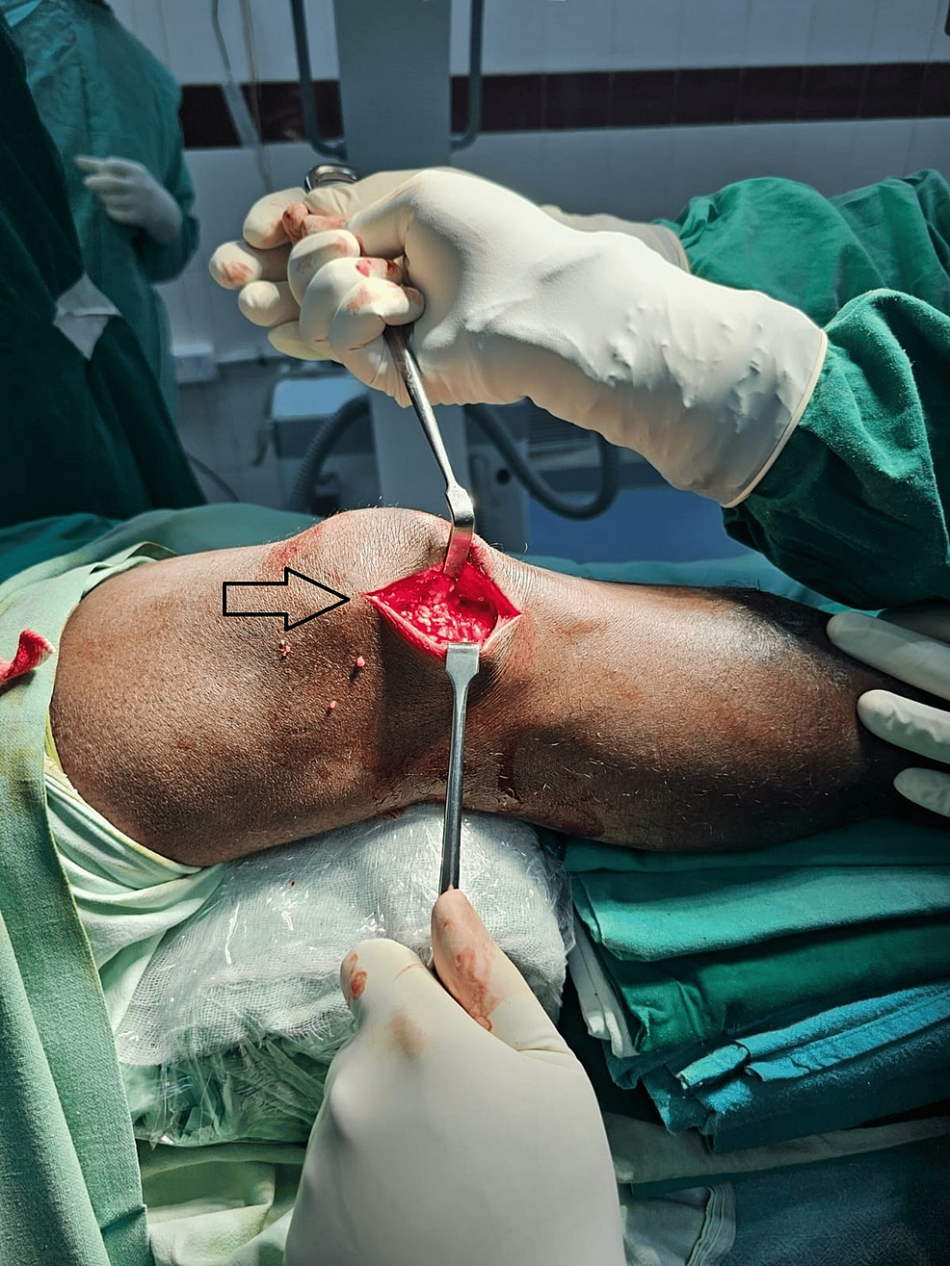
The image showing lateral retinaculum and infra-patellar fat pad.

Careful blunt dissection was performed until reaching the lateral retinaculum, which was then meticulously opened, leaving 1.5-2 cm of the retinaculum attached to the patella (Figure 2).

**Figure 3:**
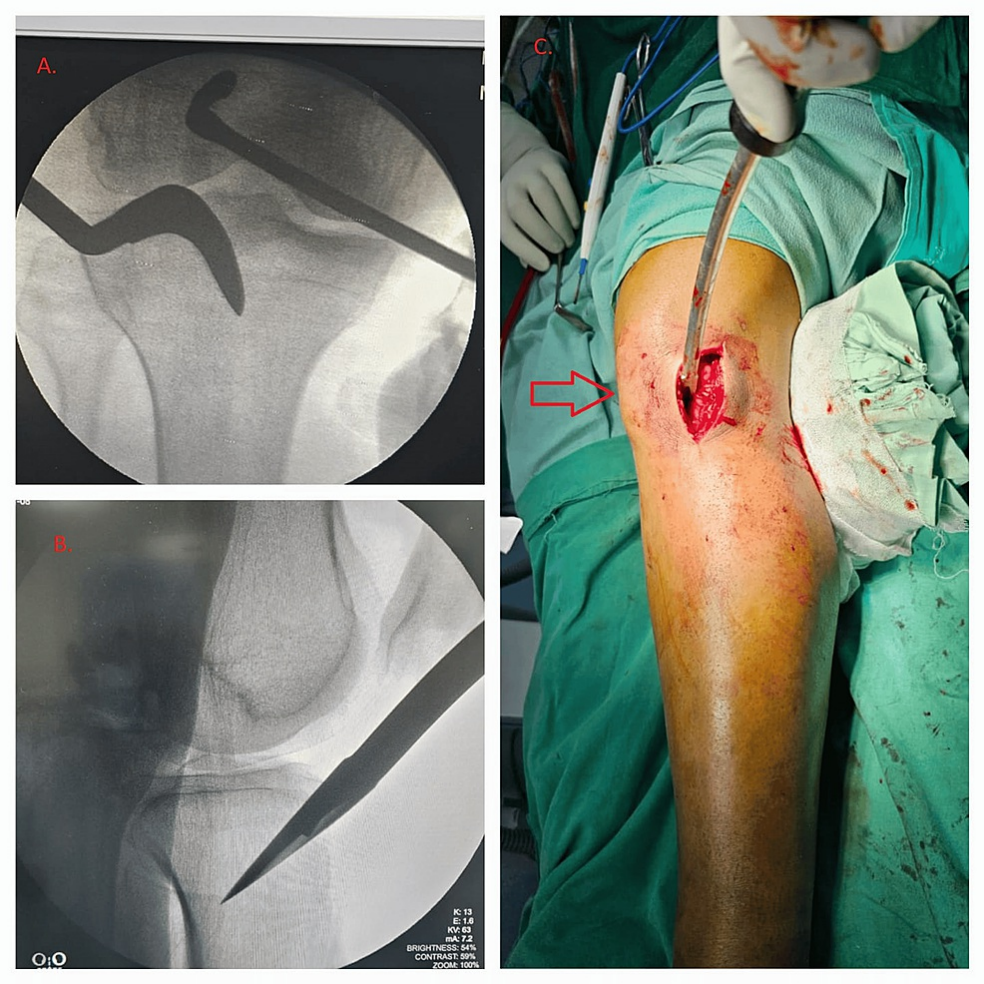
Verification of entry point using an awl under fluoroscopic guidance. (A) Anteroposterior view; (B) lateral view; (C) patella subluxated medially for making entry using an awl.

Dissection continued towards the infra-patellar fat pad, followed by medial retraction of both the fat pad and the patella. Under fluoroscopic guidance, the entry point was identified using an awl, and its position was verified on both anterior-posterior and lateral views (Figure 3).

**Figure 4:**
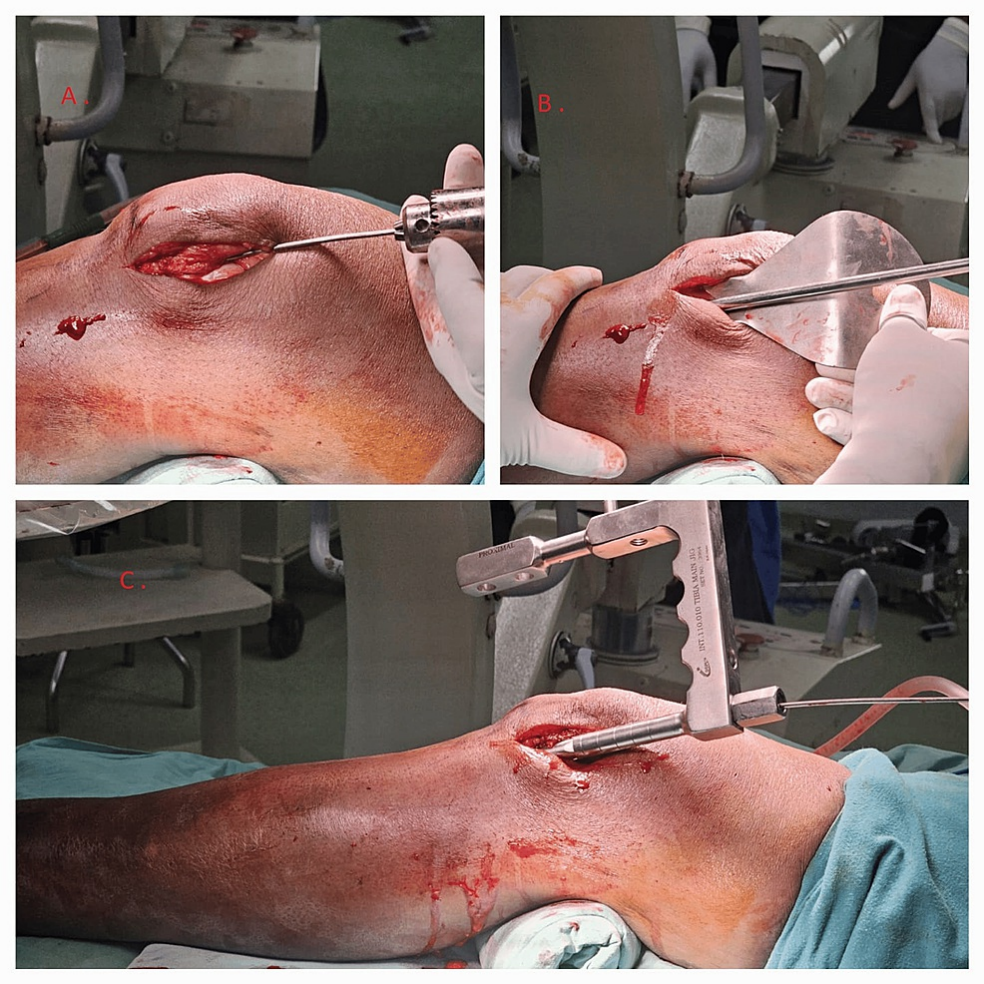
The image showing guide-wire introduction and reaming followed by tibia interlocking nail insertion through lateral para-patellar approach in a semi-extended position. (A) Guide-wire being introduced through the entry site and passed through the fracture site after closed reduction. (B) Serial reaming done with tissue protector in place. (C) Appropriate size intramedullary tibia interlocking nail inserted in semi-extended position.

The guide wire was introduced, and then closed reduction was achieved in all cases, following which the guide wire was passed through the fracture site. A tissue protector was placed, and reaming with caution to the intra-articular tissue was done (Figure 4).

**Figure 5:**
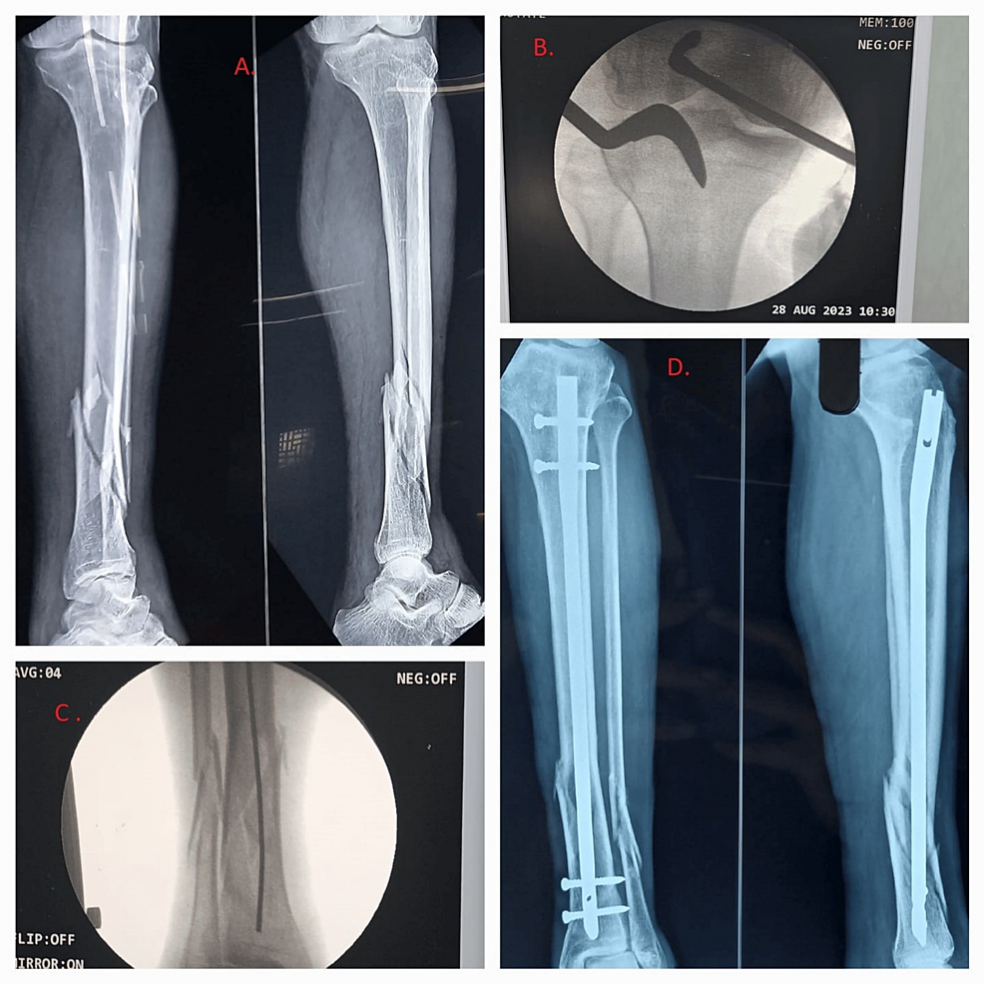
A 52-year-old male patient having distal tibia extra-articular comminuted fracture underwent tibia intramedullary nailing through lateral para-patellar approach in a semi-extended position. (A) Pre-operative radiograph showing distal tibia extra-articular comminuted fracture (AO/OTA 43-A3). (B) Anteroposterior view of entry being done with an awl passed through the lateral para-patellar approach. (C) Guide wire being passed through fracture site with closed reduction done in semi-extended position. (D) Immediate post-operative radiograph showing mild angulation. AO/OTA: Arbeitsgemeinschaft für Osteosynthesefragen/Orthopedic Trauma Association

Measurements of the nail length were taken, and then the appropriate size nail was placed. Proximal and distal locking screws were placed (Figure 5).

After nail insertion, the knee wound is irrigated to remove any reaming material, and the retinaculum is repaired with interrupted re-absorbable suture. In the postoperative period, patients are subjected to early active and passive knee and ankle mobilization and quadriceps strengthening exercises.

## Results

Sixty patients participated in the study, comprising 40 males (66.6%) and 20 females (33.3%) with a mean age of 36 (range: 18-60) years. Thirty-seven patients (61.66%) were treated for a right-sided fracture and 23 (38.33%) for a left-sided distal tibia extra-articular fracture.

Motor vehicle accident was the most common mode of trauma in our series followed by fall from height and sports injuries. Fifteen patients were smokers, six were alcoholics, and seven had diabetes. There were 38 cases of 43-A1, 14 cases of 43-A2 and eight cases of 43-A3 type of distal tibia fractures according to AO/OTA classification.

Of the 60 fractures, 54 were associated with fibular fractures, of which 39 were treated conservatively, 10 with an intramedullary wire, and five with open reduction and internal fixation. In all cases, the implants were well buried into the proximal tibia with a mean nail-apex distance of 20 mm (±6.1).

The average surgical duration was 54 ± 5 minutes, with an average of 48 intraoperative radiographic images taken (range: 38-58). No complications occurred during surgery or follow-up, and radiographic assessments confirmed satisfactory tibia nail positioning except for two cases showing mal-alignment (angular deformity <10 degrees). Fractures took an average of 16 ± 3 weeks to heal following thorough clinical and radiological evaluations.

After six months of follow-up, we observed a mean knee ROM of the treated leg of 130.6 degrees (±7.8). The contralateral mean knee ROM was 131.1 degrees (±7.2). All the evaluated knees showed full extension.

Regarding functional outcome, we observed 51 patients with excellent results on the Knee Society Score, eight with good, and one with a fair result on six months follow-ups. The mean Knee Society Score was 86.4 (±3.5).

After six months of follow-up, the mean VAS score for anterior knee pain was 0.6 (±1.1). Among the patients, 45 reported no pain, 11 experienced occasional and slight pain during intense exertion, and four reported significant pain during severe exertion. According to the Oxford scale, quadriceps strength was 5/5 in 52 patients, 4/5 in seven patients, and 3/5 in one patient. Patients' characteristics and the study outcomes have been summarized in Table 1.

**Table 1:** Patients' characteristics and their outcomes at six months follow-up. AO/OTA: Arbeitsgemeinschaft für Osteosynthesefragen/Orthopedic Trauma Association; VAS: visual analogue scale; n: number of cases

Characteristics	Patients (n=60)
Male	40 (66.6%)
Female	20 (33.3%)
Mean Age	36 years (18-60)
Fracture Classification (AO/OTA)	n (%)
43-A1	38 (63.3%)
43-A2	14 (23.3%)
43-A3	8 (13.3%)
Mode of Injury	Motor Vehicle Accidents > Fall from Height > Sports Injury
Mean Nail-Apex Distance (±S.D.)	20 mm (±6.1)
Average Surgical Duration (±S.D.)	54 (±5) minutes
Average Intra-operative Radiographic Images (range)	48 (38-58)
Average Fracture Healing Duration (±S.D.)	16 (±3) weeks
Mean Knee Society Score (±S.D.)	86.4 (±3.5) out of 100
Excellent (80-100)	51 (85%)
Good (70-79)	8 (15%)
Fair (60-69)	1 (1.6%)
Mean VAS score (±S.D.) for anterior knee pain	0.6 (±1.1) out of 10
Quadriceps Strength	n (%)
Oxford 5/5	52 (86.6%)
Oxford 4/5	7 (11.6%)
Oxford 3/5	1 (1.6%)

## Discussion

In the early 1990s, the semi-extended technique emerged as a treatment method for proximal tibial fractures. Tornetta and Collins were pioneers in recognizing that the semi-extended position effectively counters the muscular displacing forces that affect the proximal tibia during traditional flexed-knee IMN [12]. However, the initial technique's joint exposure limitations restricted its application primarily to diaphyseal tibial fractures [13,15]. Recently, advancements in nailing systems have expanded the technique's indications to include both proximal and distal tibial fractures, sparking renewed interest [17,18]. In 2010, Kubiak et al. introduced an extra-articular modification of the technique using a medial or lateral para-patellar approach [15].

Unlike the traditional knee-flexed technique, in this approach, the injured limb remains semi-extended throughout the entire procedure, offering substantial benefits to the surgeon. The improved positioning of the C-arm parallel to the floor is notably advantageous, as it simplifies the acquisition of clear and consistent images in both anteroposterior (AP) and lateral (lat) views without any interference between the C-arm and the surgical field or instruments [19].

Patient positioning is critical as it significantly impacts surgical progress. The semi-extended position reduces the need for repeated closed reductions and enhances accessibility to the entry point, resulting in reduced operation time and radiation exposure. Reviewing our centre's database revealed that, on average, 110 fluoroscopic images were used for tibia nailing via the infra-patellar approach, with an average operation time of 75 minutes. In our case series, the average surgery duration was 54±5 minutes, and the average intraoperative radiograph was 48 images, ranging from 38 to 58 images. Research by Williamson et al. and Franke et al. emphasized the advantages of the semi-extended knee position, similar to the approach utilized in this study, in reducing fluoroscopic time [20,21].

The lateral para-patellar approach appears to reduce the occurrence of anterior knee pain, which is prevalent in almost 50% of cases with traditional knee-flexed procedures [22]. While Vaisto et al. [23] and Toivanen et al. [24] found no significant difference in the incidence of anterior knee pain between para-tendinous and trans-tendinous techniques; a recent study by Rothberg et al. [25] involving 18 patients treated with this approach did not observe an increased incidence of this complication compared to a non-injured control group. Our findings indicate a minimal incidence of anterior knee pain, as evidenced by a mean VAS score of 0.6. This favourable outcome may be attributed to the nature of the technique, which minimizes disruption to the articular environment and the patellar tendon, and avoids involvement of the infra-patellar branch of the saphenous nerve.

Studies have shown that the para-patellar approach preserves knee function, as evidenced by excellent outcomes reported by various researchers. Weil et al. [26] and Rothberg et al. [25] documented good or excellent functional results in their patient series at one year of follow-up. Our findings corroborate these results, demonstrating excellent outcomes in terms of knee functional recovery after six months of follow-up. The mean Knee Society Score was 86.4 out of 100, indicating nearly complete restoration of knee ROM compared to the contralateral knee in all patients. Importantly, no patient exhibited residual knee instability, underscoring the minimal impact of this technique on intra-articular structures.

We consider the para-patellar approach to have distinct advantages over the supra-patellar approach. It is an extra-articular procedure that avoids the theoretical risk of joint injuries from the placement of working portals inside the knee [27]. Zamora et al. [28] conducted a cadaver study involving 10 specimens, demonstrating that the supra-patellar technique led to cartilage injury in the patello-femoral joint in one-third of cases, whereas the para-patellar approach did not result in significant cartilage damage. Unlike the supra-patellar method, the para-patellar approach utilizes standard equipment similar to the infra-patellar approach.

**Figure 6:**
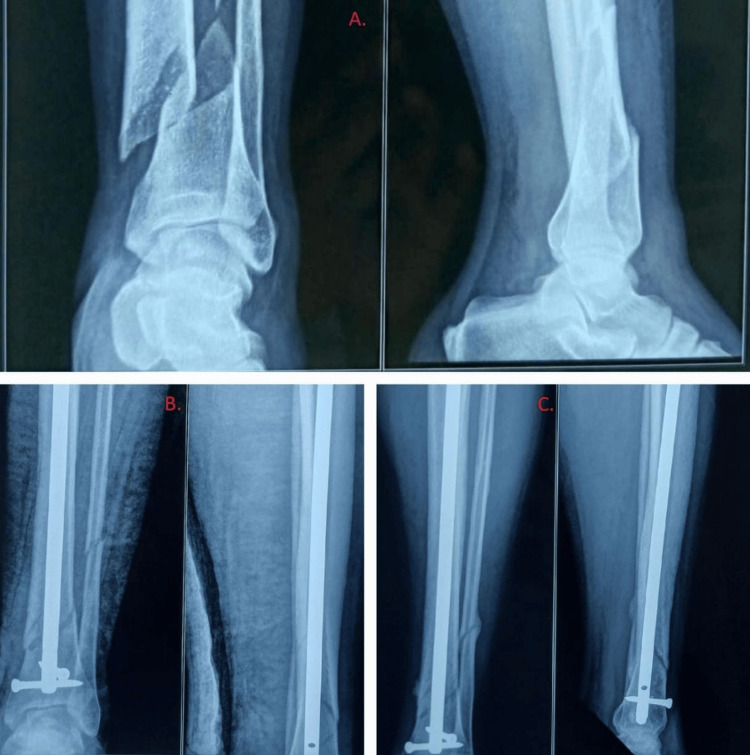
Case 1 (A) Pre-operative radiograph in anteroposterior (AP) and lateral view showing distal tibia extra-articular fracture (AO/OTA 43-A1). (B) Immediate post-operative radiograph in AP and lateral view showing fracture reduction and fixation with distal tip hole interlocking nail. (C) Three months post-operative radiograph in AP and lateral view showing callus formation with no loss of alignment and angulation. AO/OTA: Arbeitsgemeinschaft für Osteosynthesefragen/Orthopedic Trauma Association

**Figure 7:**
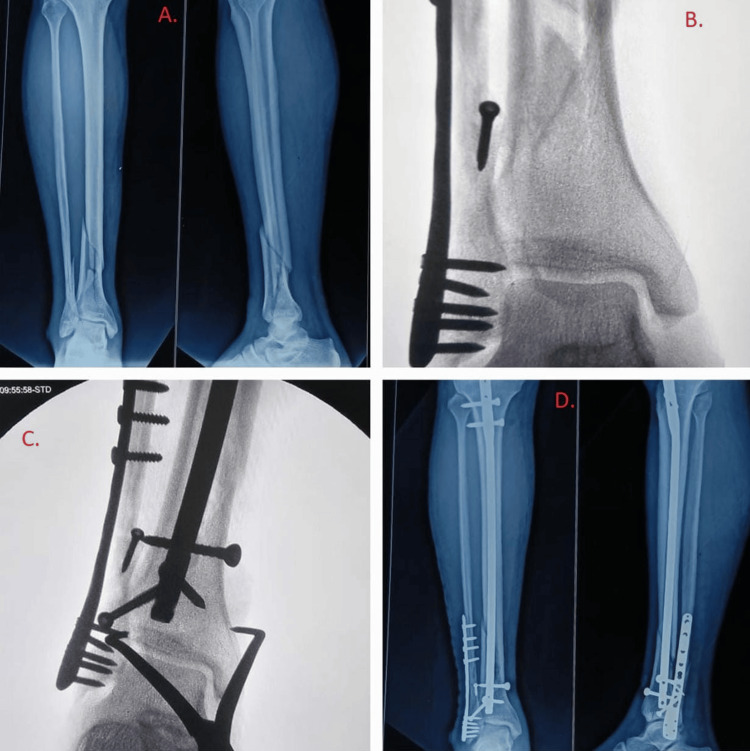
Case 2 (A) Pre-operative radiograph in anteroposterior (AP) and lateral views showing AO/OTA 43-A3 type distal tibia extra-articular fracture associated with distal fibula fracture requiring fixation. (B) Distal fibula fracture reduced and fixed first with distal fibula anatomical plate and inter-fragmentary screw. (C) Distal tibia fracture reduced and fixed with an intramedullary nail through lateral para-patellar approach with closed reduction done with the help of pointed reduction clamps in a semi-extended position of the limb. (D) Immediate post-operative radiograph on AP and lateral views showing a proper alignment of the tibia and minimal angulation at the fracture site. AO/OTA: Arbeitsgemeinschaft für Osteosynthesefragen/Orthopedic Trauma Association

While some studies propose a medial para-patellar approach for tibia INM, it was noted that this approach may heighten the risk of lateral patellar instability. Ladurner et al. outlined a similar approach [29], but in this series, a more proximal incision was chosen to facilitate easier access to the entry point while minimizing soft tissue tightening during insertion. Preservation of the lateral retinaculum rim aided closure and promoted healing, with a cautious approach to avoid sharp dissection in this area (Figures 6-7 in the Appendices).

The biggest limitation of this study is the lack of a control group of alternate techniques. Other limitations include a relatively short follow-up duration, the requirement of technical expertise, and the absence of precise radiation dose measurements.

## Conclusions

We advocate for the extra-articular lateral para-patellar approach in semi extended position for IMN due to its benefits in terms of patellar displacement, fracture reduction, reduced reliance on intraoperative radiographs, and decreased operative time, all without compromising patellar stability or worsening anterior knee pain post-operatively for distal tibia extra-articular fractures.

For future research, we advocate for randomized controlled trials with larger sample sizes to compare various tibia nailing techniques. These studies should focus on assessing their impact on key outcomes discussed in our investigation, namely operative time, radiation exposure, and anterior knee pain.

## Appendices

Case 1

A 34-year-old male with 43-A1 type fracture underwent closed reduction and internal fixation with distal tip hole interlocking tibia nail in semi-extended position through lateral para-patellar approach showing signs of union and no loss of alignment at three months follow-up (Figure 6).

Case 2

A 29-year-old male with 43-A3 type distal tibia fracture associated with distal fibula fracture, reduced and fixed with distal fibula plate and inter-fragmentary screw first, followed by IMN for distal tibia fracture through lateral para-patellar approach (Figure 7).
